# The Use of Ropivacaine in Therapeutic Treatment of Oral Aphthosis

**DOI:** 10.1155/2018/1868254

**Published:** 2018-03-11

**Authors:** Giulio Gasparini, Gianmarco Saponaro, Daniela Gasparini, Enrico Foresta, Camillo Azzuni, Alessia Adduci, Roberto Boniello, Alessandro Moro, Paolo De Angelis, Francesco Di Nardo, Giuseppe Damato, Piero Doneddu, Mattia Todaro, Umberto Garagiola, Sando Pelo

**Affiliations:** ^1^UOC Maxillo Facial Surgery, Policlinico Gemelli Foundation, Catholic University Medical School, Rome, Italy; ^2^Department of Anesthesiology and Intensive Care Medicine, Policlinico Gemelli Foundation, Catholic University Medical School, Rome, Italy; ^3^Institute of Public Health, Policlinico Gemelli Foundation, Catholic University Medical School, Rome, Italy; ^4^Department of Biomedical, Surgical and Oral Sciences, School of Dentistry, University of Milan, Milan, Italy

## Abstract

The use of anaesthetic drugs in the treatment of oral aphthosis is one of the pharmaceutical possibilities that a doctor can use for the most painful forms. Normally, Lidocaine or Diclofenac is used to treat this disease, but they can be used for a very limited time and so they are of little practical use. In this study, the authors have used Ropivacaine whose pharmaceutical kinetics allows the analgesic effect to be active for 60 to 90 minutes. In our research, we compared 8 groups of patients who have been given 3 principal pharmaceutical products: one group was given an anaesthetic drug, one had a topical medication administered which is often used for the treatment of aphthous lesions, and the last group was given a multivitamin. These pharmaceutical products were used alone and in various possible combinations in the 8 groups. The results of this study are very interesting and show that in all the groups that used anaesthetics there was more satisfaction on the patients' part because their pain level became more manageable right after the first application of the drug and the patients could carry on with their normal lives.

## 1. Introduction

The aphtha is a lesion of the oral mucous tissue characterized by the presence of a very painful ulcer. It can be found alone or it can occur in groups in several different places in the oral cavity. At the beginning it gives the patient a feeling of itchiness or a burning sensation in the mouth where a lesion will appear in one or two days. The aphtous ulcer has an oval form and has a diameter from a minimum of 3 to 4 mms to a maximum of 10 to 15 mms. Its floor color is a white or yellow, and around it is an area which is extremely hyperemic due to the inflammation taking place which can cause submandibular lymph nodes swelling and pain. The pain can extend to the dental arch and is often confused with toothache. As days pass, a whitey-grey spot is formed which consists of fibrin deposits [1, 2].

Aphthae affect from 5 to 25% of the population according to different studies and various methodologies. This disorder is more frequently found in women and in the higher social classes [3–5]. The region of the oral cavity that is most often affected is the mucosa of the lower lip, the tongue, the retromolar trigone on the buccal gums, the palate, the throat, and the larynx [1–5].

The length of the illness varies from patient to patient. It can vary between 7 and 15 days when there are single aphthae, but the illness can go on for up to a month if there are multiple aphthae. In the most serious cases a scar can form where the lesion was, but this tends to disappear in time. The aphthae are not contagious, and a differential diagnosis has to be made considering simple herpes, other autoimmune diseases such as Vulgaris Pemphigus, viral diseases, candidiasis, ACLE (acute cutaneous lupus erythematosus), Allergic Contact Dermatitis, Cancers of the Oral Mucosa, Cicatricial Pemphigoid, Contact Stomatitis, Mucosae Aspects of Behcet's Disease, Oral Mucosae Manifestations of Gastrointestinal Disease, Oral Mucosae Manifestations of Hand-Foot-and-Mouth Disease, Oral Mucosae Manifestations of Hematologic Disease, Drug-Induced Bullous Disorders, Drug-Induced Lupus Erythematosus, Drug-Induced Pemphigus, Erythema Multiforme, IgA Pemphigus, Irritant Contact Dermatitis, Langerhans Cell Histiocytosis, Lichen Planus, Linear IgA Dermatosis, Paraneoplastic Pemphigus, and Pediatric Syphilis [6, 7]. The aphthous lesions are not caused by a virus. The causes are not well known even though it seems likely that it is a disorder of the bacterial flora in the oral cavity associated with immunity problems. Among the factors that raise the risk of developing aphthae are contact of the mucosal membrane with dirty objects and domestic animals [1]. Aphthae can be caused by many situations among which are stress, poor oral hygiene, disorder of the bacterial flora in the intestine, immune deficiency, lack of iron and zinc, and lack of vitamins from the “B” group [1, 2, 8–10].

Several studies have implicated vitamin deficiencies (B12, folic acid, B1, B2, and B6) in the pathogenesis of recurrent aphthous stomatitis [11–18]. Although this topic is controversial due to lack of evidence, the role of vitamin deficiencies is recognized, especially vitamin B12, as a possible predisposing factor. In patients affected by vitamin deficiencies a specific replacement therapy may help to correct the deficiency and improve the patient's condition or induce remission [18].

Some studies show that some people are more genetically prone to the aphthae because of certain antigens in the Major Histocompatibility [1, 2]. Many Chronic diseases are related to the appearance of aphthae: Ulcerative Colitis, Crohn's Disease, Bullous Pemphigoid, Leukoplakia, Behcet's Disease, Lichen Planus,* Candida albicans*, and Coeliac Disease. Some food intolerances can also be the cause of aphthae like, for example, the excessive consumption of chocolate and strawberries [1].

For people who are prone to getting aphthae, there are many situations that can cause them. For example, accidental bites of the mucosal tissue, too hard brushing of the mucosal tissue, or little lesions provoked by food can cause this problem. Often the original lesion has an abundance of floral bacteria in it that causes the involvement of the lymph nodes, which is a sign that often accompanies aphthae in the mouth. Aphthous lesions are very painful and are often associated with a general sickly feeling, and in some cases fever accompanies this feeling. When there are multiple aphthae, it is often difficult to eat due to the impossibility of chewing food and the pain that comes from the scraping of the food against the lesion. When the aphthae are located near the soft palate or esophagus, it becomes difficult to swallow due to odynophagia. The lip or tongue aphthae make it difficult to talk, but this is not due to mechanical problems caused by the aphthae, but it is the fear and the pain that cause the patients to limit their speech. In order to reduce the pain from this disorder, we wanted to add an anaesthetic drug to the normal therapy for aphthous lesions in order to observe if the satisfaction of the patient improved and also to see if the length of the disease's appearance can be shortened.

## 2. Materials and Methods

To carry out this study we selected 80 patients (table of patients (available here)) referred to our attention for different reasons. The inclusion criteria were that the patients had at least 3 episodes of aphthae within the year, their age was over 18 years, and they were not affected by other diseases of the oral cavity and the gastrointestinal tract. The study lasted 12 months from the June 1, 2015, to June 1, 2016. This research was conducted in private practice; thus it did not require approval by the local ethics committee. The investigators were trained about study-specific methods, and university researchers have made a contribution only to data entries and to evaluation of the clinical progress. All investigations reported have been carried out in accordance with the 1975 Helsinki Declaration, as revised in 2013 for ethical approval. All participants provided written informed consent after receiving explanations on study objectives and procedures. We asked the patients to begin the treatment with medicines we proposed at the first moment the aphthae symptoms appeared. The last day of treatment was when the patient felt no more need to follow the therapy. Four parameters were considered:

- Pain was graded according to an analytical scale of 0 to 5 where 0 represents normality and 5 is the value given to the highest pain possible.
- Difficulty in eating (intended as the difficulty in keeping food or liquids in the oral cavity as well as the difficulty in chewing) was graded according to an analytical scale where 0 represents normality and 5 represents the impossibility of eating.
- Difficulty in swallowing (intended as normal swallowing up to impossibility to swallow at all) was graded according to an analytical scale where 0 represents normal swallowing and 5 represents the top level of swallowing difficulty.
- Difficulty in producing sounds or speech again is represented in analytical scale with 0 representing the normal ability to produce sounds and 5 representing the impossibility to talk at all.

With each group we considered the number of relapses that each patient had, the type of aphthae on each patient (by the type of aphthae we mean whether they were single aphthae or multiple aphthae), and where these aphthae were found in the oral cavity.

Groups were formed in a random way by extracting names from an urn.

We formed the following groups:

- *Placebo group (P)*: this group was given vaseline oil.
- *Anaesthetic group (A)*: the active ingredient that we gave and that reacted on contact with the aphthae was Ropivacaine 100 mgs in 10 mls: the patient was told to put the anaesthetic on the lesions with a cotton pad and hold it there for 10 seconds.
- *Pharmaceutical drugs group (M)*: this group was given a medicinal compound already present in the market based on Sorbitol Aqua, Propanediol (Zemea), Aqua, Propylene Glycol, Polycarbophil (Noveon AA-1USP), Mucosave FG (Maltodextrin, Opuntia Ficus-Indica stem extract, Olea Europaea leaf extract), Sodium Hydroxide, Taurine, PVP (Luviskol K90), Geogard Ultra (Gluconolactone, Sodium Benzoate, Calcium Gluconate), Xylitol, Sodium Hyaluronate, SymRelief (Bisabolol, Zingiber Officinale root extract), Sucralose, Aroma, and Stevia Rebaudiana extract. This medicinal compound is an alcohol-free gel which was applied 2 to 3 times a day or more if needed after the meals during one week up to complete removal of the symptoms.
- *Vitamin group (V)*: this group was given a pharmaceutical compound already in the market based on vitamin A, D, E, B1, B2, B6, B12, C, E, folic acid, pantothenic acid, biotin, niacin, calcium, magnesium, iron, copper, iodine, zinc, manganese, and molybdenum. The administration of these vitamins was the giving of 1 pill a day from the moment that the disease became evident taken up to 7 days after the end of the disease [10, 19, 20].
- *Vitamin + local anaesthetic + pharmaceutical drugs (VAM)*: this group was given the three medications described in the other preceding groups in the same way as they were introduced to the other groups, except that they were given in a combined way. First the anaesthetic was given, then medicine was given, and, after the anaesthetic took effect, the vitamin pill was administered.
- *Vitamin + pharmaceutical drugs (VM)*: this group was given the two medications described in the previous groups and they were given in the order already described: first the pharmaceutical medicine was given and later the vitamin.
- *Anaesthetic + pharmaceutical drugs (AM)*: these medications were given in the same way as they were given in the other groups with the anaesthetic given first and then the other medicine given after the anaesthetic took effect.
- *Anaesthetic vitamin group (AV)*: this group was given the two medications. As in the other groups, the anaesthetic was administered first, and then the vitamin pill was given after the anaesthetic took effect.

### 2.1. Statistical Analysis

**Figure 1:**
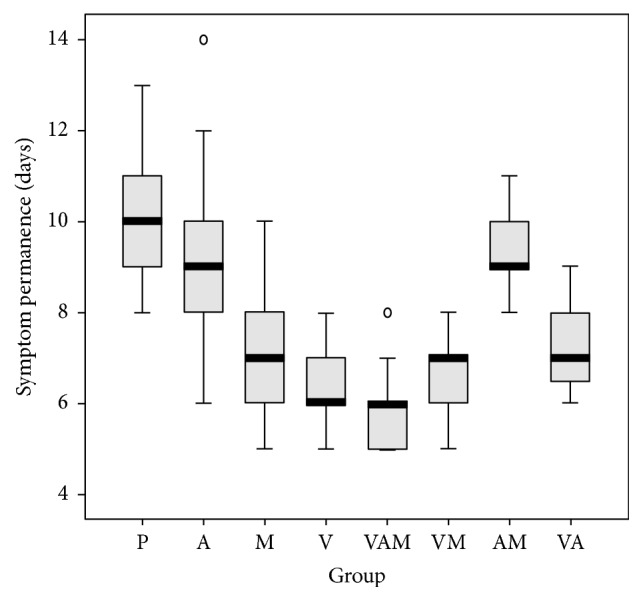
Symptoms duration (days) by treatment. Box plot. Black bar: median value. Box: interquartile range. Whisker: last value within 1.5 times the interquartile range from the median. Circles: outliers with a value between 1.5 and 3.0 times the interquartile range from the median. Asterisks: outliers with a value over 3.0 times the interquartile range from the median. V: treatment with vitamins; A: treatment with anesthetics; P: treatment with drugs. Kruskal Wallis test: *p* < 0.001.

**Figure 2:**
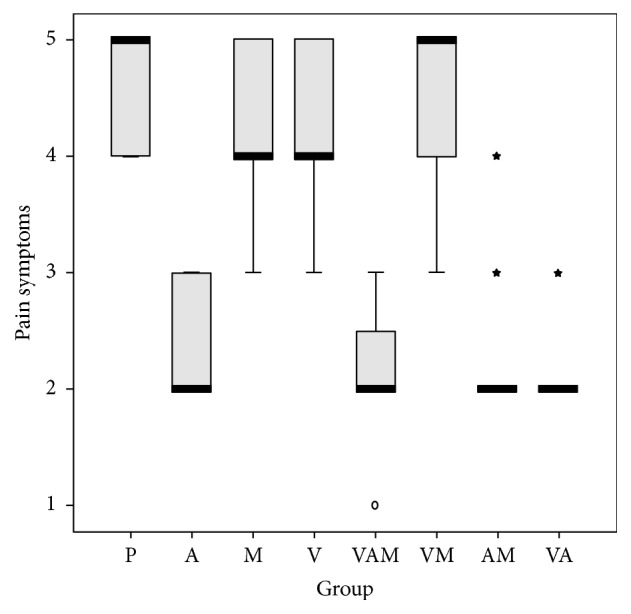
Pain on a scale from 1 to 5 by treatment. Box plot. Black bar: median value. Box: interquartile range. Whisker: last value within 1.5 times the interquartile range from the median. Circles: outliers with a value between 1.5 and 3.0 times the interquartile range from the median. Asterisks: outliers with a value over 3.0 times the interquartile range from the median. V: treatment with vitamins; A: treatment with anesthetics; P: treatment with drugs. Kruskal Wallis test: *p* < 0.001.

**Figure 3:**
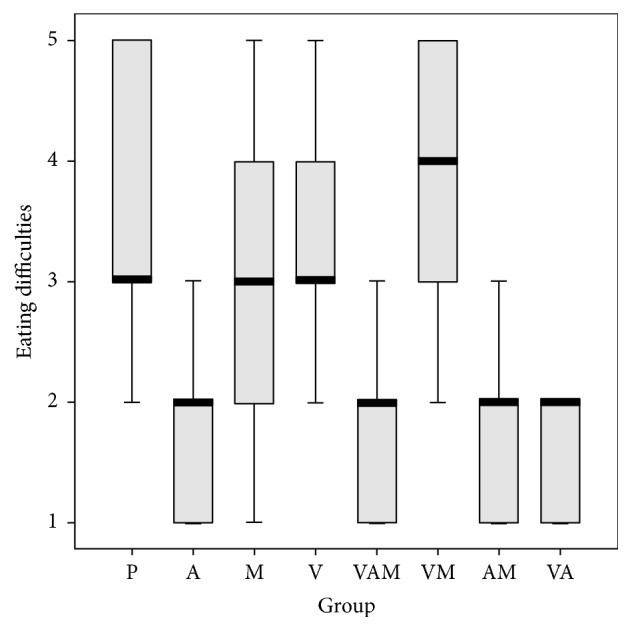
Eating difficulties on a scale from 1 to 5 by treatment. Box plot. Black bar: median value. Box: interquartile range. Whisker: last value within 1.5 times the interquartile range from the median. Circles: outliers with a value between 1.5 and 3.0 times the interquartile range from the median. Asterisks: outliers with a value over 3.0 times the interquartile range from the median. V: treatment with vitamins; A: treatment with anesthetics; P: treatment with drugs. Kruskal Wallis test: *p* < 0.001.

**Figure 4:**
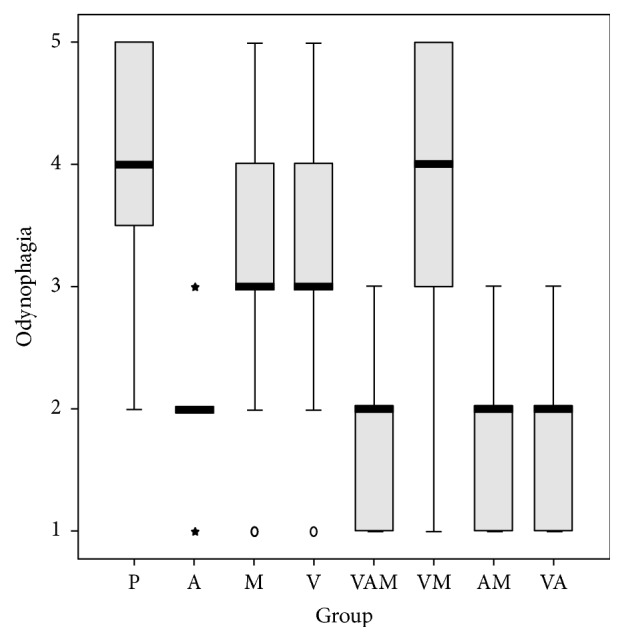
Pain while eating on a scale from 1 to 5 by treatment. Box plot. Black bar: median value. Box: interquartile range. Whisker: last value within 1.5 times the interquartile range from the median. Circles: outliers with a value between 1.5 and 3.0 times the interquartile range from the median. Asterisks: outliers with a value over 3.0 times the interquartile range from the median. V: treatment with vitamins; A: treatment with anesthetics; P: treatment with drugs. Kruskal Wallis test: *p* < 0.001.

**Figure 5:**
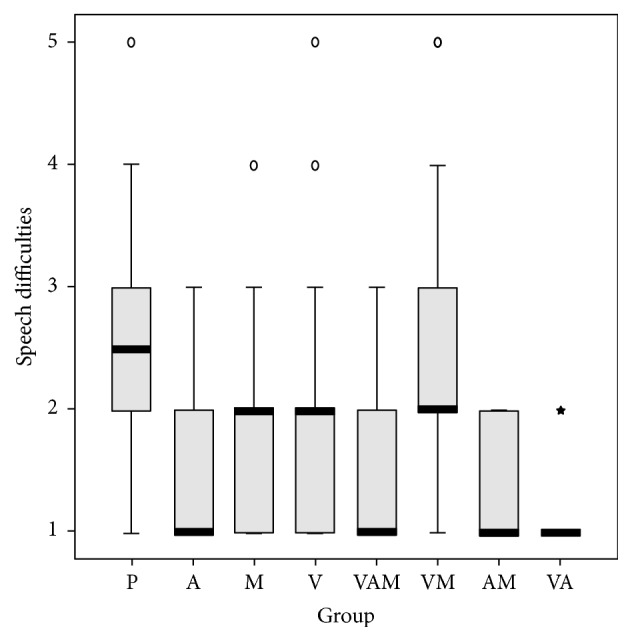
Speech difficulties on a scale from 1 to 5 by treatment. Box plot. Black bar: median value. Box: interquartile range. Whisker: last value within 1.5 times the interquartile range from the median. Circles: outliers with a value between 1.5 and 3.0 times the interquartile range from the median. Asterisks: outliers with a value over 3.0 times the interquartile range from the median. V: treatment with vitamins; A: treatment with anesthetics; P: treatment with drugs. Kruskal Wallis test: *p* < 0.001.

We conducted a logistic regression to determine the effect of the anaesthetic, vitamins, and medicines. We studied their effects on lesion duration, pain, eating difficulties, swallowing, and difficulties in speech (Figures 1–5). In the logistic regression models data has been corrected for age, sex, and location of the aphthae (either single or multiple aphthae). For all the analyses only *p* values ≤ 0.01 were considered statistically significant. The coefficients of each covariate are reported with the relative intervals of confidence at 95% (I.C. 95%). The analysis was conducted with SPSS software version 13.0 for windows.

## 3. Results

80 patients have been selected for this study: 36 females and 44 males between the ages of 19 and 65 with the mean age being 39,5 years. 

*P Group*. It was made up of 7 males and 3 females between the ages of 18 and 41 with the average age being 31,8 years. In this group we had 48 episodes of aphthae with an average of 4,8 episodes of aphthae per patient. In total we had 13 episodes of single aphthae and 35 episodes of multiple aphthae with an average of 1,3 for single aphthae and 3,5 for multiple aphthae episodes per patient. The length of these episodes was from 8 to 13 days with the average length being 10,2 days. The pain symptom varied between 2 to 5 days with the average being 3,5 days. The parameters for swallowing difficulties varied from 1 to 3 days with the average number of days being 2,2 days of swallowing difficulties. The parameter for speech difficulties varied from 1 to 5 days with the average being 2,5 days of difficulties in speaking. 

*A Group.* It was composed of 6 males and 4 females between the ages of 23 and 63 years. The mean age was 47,2 years. In this group there was an average of 4,5 episodes per patient. In this group we had 20 single aphthae and 25 multiple aphthae with an average of 2 for single aphthae and 2,5 for multiple aphthae episodes per patient. The average length of the episodes varied from 6 to 14 days with the mean length of 8,9 days of illness. The pain symptom varied from 2 to 3 days with the average being 2,4 days of pain. Eating difficulties varied in length from 1 to 3 days with the average length being 2,2 days of pain. Speech difficulties varied between 1 and 3 days with the average being 1,5 days of difficulty in speaking. 

*M Group*. It was made up of 7 males and 3 females aged between 23 and 63 years, withthe average age being 47,2 years. In this group we had a total of 50 aphthous episodes with the average of 5 episodes per patient. In total we had 22 episodes of single aphthae and 28 episodes of multiple aphthae with an average of 2,8 and 2,2 episodes, respectively, per patient. The average length of these episodes lasted from 5 to 10 days with the average duration being 7,2 days. The pain symptoms varied between 3 and 5 days with the average being 4 days of pain. The parameters of eating difficulties varied from 1 to 5 days with the average length of time being 2,9 days. The swallowing problems varied in length from 1 to 5 days with the average length of time of swallowing difficulties being 3,4 days. Speaking difficulties varied in length between 1 and 4 days with the average being 1,9 days. 

*V Group*. It was made up of 6 males and 4 females between the ages of 18 and 56 with the average age being 36,6 years. In this group we had a total of 47 episodes of aphthae, with the average number of episodes per patient being 4,7. Altogether we had 23 episodes of single aphthae and 24 episodes of multiple aphthae, and the average number of aphthous episodes per patient was 2,3 and 2,4 episodes, respectively, per patient. The average length of the illness was from 5 to 8 days, with the average length being 6 days. The pain symptom lasted between 3 to 5 days with the average length in time being 4,4 days of pain. The parameters evaluating the difficulty in eating varied between 2 to 5 days with the average number of days being 3,4 days per patient. The parameters revealing swallowing difficulty varied between 1 and 5 days with the average number of days being 2 days. 

*VAM Group*. It was composed of 4 males and 6 females aged between 25 and 52 with average age being 36,9 years. In this group we had a total of 43 episodes of aphthae with the average of 4,3 episodes per patient. In total we had 25 episodes of single aphthae and 18 episodes of multiple aphthae with an average number of episodes being, respectively, 2,5 episodes for single aphthae and 1,8 for multiple aphthae per patient. The average length of these episodes per patient was, respectively, 5 and 8 days with an average of 6 days for each patient. The pain symptom varied between 1 and 3 days with an average of 2,2 days of pain. The parameter evaluating the length of time patients had eating difficulties varied between 1 and 3 days with the average being 1,7 days. The parameter measuring the length of time patients suffered difficulty in swallowing varied between 1 and 5 days with the average being 3,4 days per patient. Speaking difficulties varied between 1 and 3 days with the average number of days being 1,3. 

*VM Group*. It was composed of 5 males and 5 females between the ages of 28 and 64 years, with the average age being 44,3 years. In this group we had a total of 44 episodes of aphthae with an average of 4,4 episodes per patient. In total we had 26 episodes of single aphthae and 18 episodes of multiple aphthae with an average of 2,6 for single aphthae and 1,8 for multiple aphthae. The average length of the episodes for the pain symptom varied between 5 and 8 days with the average being 6,6 days. The parameter measuring eating difficulties varied between 2 and 5 days with the average number of days being 3,7 days. Speaking difficulties varied between 1 and 5 days, with the average being 2,5 days in length. 

*AM Group*. It was composed of 5 males and 5 females ranging in age between 19 and 61 with the average being 39,5 years. In this group we had a total of 44 episodes of aphthae with the average number of aphthae being 4,4. In total we had 1,6 episodes for single aphthae and 2,8 episodes for multiple aphthae. The average length of the pain symptom varied from 8 to 11 days with an average of 9,3 days of pain per patient. The parameter measuring difficulty in eating varied from 1 to 3 days with the average number of days being 1,8 days. Swallowing difficulties varied between 1 and 3 days too with the average length of time being 1,8 days as well. Speaking difficulties varied in length between 1 and 2 days with an average of 1,3 days per patient. 

*AV Group*. It was made of 4 males and 6 females between the ages of 19 and 65 with an average of 39,4 years. In this group we had a total of 43 episodes of aphthae with the average number of episodes being 4,3 episodes per patient. In total we had 22 single aphthae and 21 multiple aphthae episodes with the average being, respectively, 2,2 and 2,1 episodes. The average length of these aphthae episodes varied between 6 and 9 days with an average of 7,2 days. Pain symptom from these aphthae lasted from 2 to 3 days with the average length of pain duration being 2,1 days. Eating difficulties varied from 1 to 2 days in duration with the average being 1,6 days. Swallowing problems lasted from 1 to 3 days with the average length of time being 1,7 days. Speaking difficulties varied in duration from 1 to 2 days with an average duration of 1,2 days.

Vitamins reduced the length of the appearance of the aphthae to 2,2 days on average (IC 95%; 2,5–2,0, *p* ≤ 0.001) while the topical medicine reduced the length of the disease by 0,8 days (IC 95%: 1,1–5,6, *p* < 0.001). The appearance of multiple aphthae took longer to be cured (+0,8 days, IC 95%: 0,6–1,1, *p* < 0.001).

The use of anaesthetics proved to significantly lower the values of pain (−2,2 points, IC 95% 2,3–2,1, *p* < 0,001, Table 2). The presence of multiple aphthae is significantly linked to greater pain (+0,4 points, IC 95%: 0,3–0,5, *p* < 0,001).

The use of anaesthetics proved to be equally and significantly linked to lower values on the scale measuring eating difficulties (on average −1,7 points, IC 95%: 1,9–1,5, *p* < 0,001); however, the appearance of multiple lesions is linked to higher values (on average +0,3 points, IC 95%: 0,1–0,5, *p* = 0,003, Table 3).

Table 4 shows the results of the multivariate analysis regarding the difficulty in swallowing. It shows that the use of anaesthetics significantly reduced the values by 1,8 pts on the analogical scale of 1 to 5 (IC 95%: 2,0–1,6, *p* < 0,001). The presence of multiple lesions and the male sex bring about a significant raise in the values, respectively, by 0,4 pts (IC 95%: 0,2–0,6, *p* < 0,001) and 0,3 pts (IC 95%: 0,1–0,4, *p* = 0,010).

Finally, the use of anaesthetics is linked to significantly lower values on the analogical scale for difficulty in speaking (−0,9 points, IC 95%: 1,1–0,8, *p* < 0,001); however, the presence of multiple aphthae brings much higher values (+0,3 points, IC 95%: 0,1–0,4, *p* = 0,002, Table 5).

## 4. Discussion

In the treatment of aphthous lesions, anaesthetics such as Lidocaine and Diclofenac have been used for some time. However the length of time these anaesthetics bring relief is brief [21, 22]. Due to this defect we decided to test the use of Ropivacaine which is an anaesthetic drug used in aphthous lesions. Ropivacaine is an amide local anaesthetic that gives anaesthetic and analgesic relief over a long period. High dosages produce anaesthetic pain blockage which is used in surgery, while low dosages, through topical application, cause a sensitive blockage with a limited motor block; thus all the essential movements of the involved muscles are saved. The topical application acts mainly by inhibiting sodium influx through sodium-specific ion channels in the neuronal cell membrane.

Consequently it reversibly decreases the rate of depolarization and repolarization of excitable membranes blocking neuroimpulses on the area where it is applied [23]. The most noteworthy feature of Ropivacaine is its long-lasting effect. Its anaesthetic qualities go into effect immediately and it lasts from 60 to 90 minutes, which allows the patient to carry out normal daily function without problems. The effect of the anaesthetic on aphthous lesions is better than the effect that anaesthetic has on normal mucosa because the drug can easily reach the nerve endings of the area involved by the lesion where it goes into action.

The role of nutritional deficiency as a cause of recurrent aphthous stomatitis has been noticed because of the association of a subset of these patients with low serum levels of iron, folate, zinc, vitamins B1, B2, B6, B12, vitamin C, and calcium [24]. This is an indication that vitamin deficiencies related to nutritional causes or to malabsorption may be an etiological factor for recurrent aphthae. There are few available controlled studies analyzing hematological status of patients with recurrent aphthous stomatitis and the results reported are controversial [24]. Furthermore studies analyzing the effect of supplemental vitamin treatments are small in size, uncontrolled, not randomised, and unmasked [19, 25]. A study of Volkov et al. reported high effectiveness of vitamin B12 administration regardless of its initial levels in the serum. Despite these results, there was a lack of any consistent biological explanation for the benefits observed with the authors suggesting an unrecognized function of vitamin B12 [24, 25].

In our study, we wanted to evaluate our patient satisfaction and the different length of the disease when combining various drugs normally used in the treatment of aphthous lesions. From what we could observe by the statistical analysis, taking vitamins together with the pharmaceutical medicine shortened the length of the illness (Table 1).

From the analysis of regressive logistics results, it comes out that while medicines and vitamins seem to have a role in reducing the length of the appearance of aphthae, the use of anaesthetics has more beneficial results on the pain, difficulty in eating, swallowing, and speaking.

## 5. Conclusions

From our research it has been proved without doubt that the use of local anaesthetics is able to improve all the symptoms caused by aphthous lesions in the oral cavity. In particular, Ropivacaine has been shown to have a rapid and long-lasting effect on the pain symptom which greatly improved the quality of life in patients suffering from aphthae. Moreover, patients who used the combined treatment of vitamins and topical drugs healed faster. The outcome of our research shows that combined therapy of anaesthetic-topical medicine and vitamins was able to give a dependable solution for patients with oral aphthosis guaranteeing less pain and less suffering from the disease and reducing the length of the appearance.

## Figures and Tables

**Table 1:** Logistic regression. Dependent variables: length in days. IC 95%: interval of confidence at 95%.

Variables	Coeff. *B*	*p*	IC 95%, lower limit	IC 95%, higher limit
Anaesthetic	0,318	0,022	0,046	0,590
Pharmaceutical drugs	−0,824	<0,001	−1,091	−0,557
Vitamin	−2,242	<0,001	−2,516	−1,968
Multiple aphthosis	0,834	<0,001	0,562	1,105
Age	0,007	0,196	−0,004	0,019
Male sex	0,159	0,257	−0,117	0,435

**Table 2:** Logistic regression. Dependent variables: pain (analogic scale from 1 to 5). IC 95%: confidence interval at 95%.

Variables	Coeff. *B*	*p*	IC 95%, lower limit	IC 95%, higher limit
Anaesthetic	−2,171	<0,001	−2,285	−2,057
Pharmaceutical drugs	−0,126	0,028	−0,237	−0,014
Vitamin	0,075	0,199	−0,040	0,189
Multiple aphthosis	0,381	<0,001	0,268	0,495
Age	0,004	0,125	−0,001	0,008
Male sex	0,056	0,338	−0,059	0,172

**Table 3:** Logistic regression. Dependent variables: eating difficulty (analogical scale from 1 to 5). IC 95%: confidence interval at 95%.

Variables	Coeff. *B*	*p*	IC 95%, lower limit	IC 95%, higher limit
Anaesthetic	−1,716	<0,001	−1,903	−1,529
Pharmaceutical drugs	−0,084	0,367	−0,268	0,099
Vitamin	0,177	0,065	−0,011	0,365
Multiple aphthosis	0,279	0,003	0,092	0,465
Age	0,006	0,127	−0,002	0,014
Male sex	−0,002	0,981	−0,192	0,187

**Table 4:** Logistic regression. Dependent variables: difficulty in swallowing (analogical scale of 1 to 5). IC 95%: confidence interval at 95%.

Variables	Coeff. *B*	*p*	IC 95%, lower limit	IC 95%, higher limit
Anaesthetic	−1,788	<0,001	−1,976	−1,600
Pharmaceutical drugs	−0,166	0,077	−0,350	0,018
Vitamin	−0,132	0,169	−,0321	0,057
Multiple aphthosis	0,384	<0,001	0,197	0,572
Age	0,007	0,102	−0,001	0,014
Male sex	0,250	0,010	0,060	0,440

**Table 5:** Logistic regression. Dependent variables: difficulty in speaking (analogical scale of 1 to 5). IC 95%: confidence interval at 95%.

Variables	Coeff. *B*	*p*	IC 95%, lower limit	IC 95%, higher limit
Anaesthetic	−0,922	<0,001	−1,086	−0,759
Pharmaceutical drugs	−0,059	0,471	−0,219	0,102
Vitamin	−0,011	0,891	−0,176	0,153
Multiple aphthosis	0,257	0,002	0,093	0,420
Age	0,008	0,022	0,001	0,015
Male sex	−0,167	0,049	−0,332	−0,001

## Supplementary Materials

Supplementary Materials Table of patients: 80 patients subdivided into 8 groups. In every patient we studied the effect of the aphthae on the quality of life. In every aphthae event we studied pain, difficulty in eating, difficulty in swallowing, and difficulty in producing sounds or speech. With each group we considered the number of relapses that each patient had, the type of aphtha on each patient, and where these aphthae were found in the oral cavity. We formed the following groups: placebo group (P), anaesthetic group (A), pharmaceutical drugs group (M), vitamin group (V), vitamin + local anaesthetic + pharmaceutical drugs (VAM), vitamin + pharmaceutical drugs (VM), anaesthetic + pharmaceutical drugs (AM), and anaesthetic vitamin group (AV).
